# Copper Deficiency in the Lungs of TNF-α Transgenic Mice

**DOI:** 10.3389/fphys.2016.00234

**Published:** 2016-06-14

**Authors:** Liu Liu, Xiangrong Geng, Joseph McDermott, Jian Shen, Cody Corbin, Stephanie Xuan, Jae Kim, Li Zuo, Zijuan Liu

**Affiliations:** ^1^Department of Biological Sciences, Oakland UniversityRochester, MI, USA; ^2^Department of Pathology, Creighton University School of MedicineOmaha, NE, USA; ^3^Radiologic Sciences and Respiratory Therapy Division, School of Health and Rehabilitation Sciences, The Ohio State University College of Medicine, The Ohio State University Wexner Medical CenterColumbus, OH, USA

**Keywords:** biometals, COPD, inflammation, micronutrients, oxidative stress

## Abstract

Tumor necrosis factor (TNF)-α is a well-known pro-inflammatory cytokine. Increased expression of *Tnf*-α is a feature of inflammatory lung diseases, such as asthma, emphysema, fibrosis, and smoking-induced chronic obstructive pulmonary disease (COPD). Using a mouse line with lung-specific *Tnf*-α overexpression (SPC-TNF-α) to mimic TNF-α-associated lung diseases, we investigated the role of chronic inflammation in the homeostasis of lung trace elements. We performed a quantitative survey of micronutrients and biometals, including copper (Cu), zinc (Zn), and selenium (Se), in the transgenic mice tissues. We also examined the expression of Cu-dependent proteins in the inflammatory lung tissue to determine whether they were affected by the severe Cu deficiency, including cuproenzymes, Cu transporters, and Cu chaperones. We found consistent lung-specific reduction of the metal Cu, with a mean decrease of 70%; however, Zn and Se were unaffected in all other tissues. RT-PCR showed that two Cu enzymes associated with lung pathology were downregulated: amine oxidase, Cu containing 3 (*Aoc3*) and lysyl oxidase (*Lox*). Two factors, vascular endothelial growth factor (*Vegf*) and focal adhesion kinase (*Fak*), related with Cu deficiency treatment, showed decreased expression in the transgenic inflammatory lung. We concluded that Cu deficiency occurs following chronic TNF-α-induced lung inflammation and this likely plays an essential role in the inflammation-induced lung damage. These results suggest the restoration of lung Cu status as a potential strategy in both treatment and prevention of chronic lung inflammation and related disorders.

## Introduction

Many pulmonary diseases feature significant upregulation of cytokines such as tumor necrosis factor (TNF)-α. For example, pulmonary fibrosis (Raghu et al., 2008), steroid refractory asthma (Berry et al., 2006), chronic obstructive pulmonary disease (COPD) (Keatings et al., 1996; Churg et al., 2002; Sakao et al., 2002), pulmonary Langerhans' cell histiocytosis (Vassallo et al., 2000), and emphysema (Lucey et al., 2002; Vuillemenot et al., 2004) all demonstrate a trend of inflammatory cytokine upregulation. Commonly adopted techniques for treating inflammatory lung diseases involve directly inhibiting inflammatory signaling and halting inflammatory positive-feedback loops (Barnes, 2013; Vettorazzi et al., 2015). Therapies targeting *Tnf*-α overexpression are currently the most utilized treatment option. However, novel strategies are needed to more effectively combat inflammation-associated lung disorders. Current treatments aim to block the progression of inflammatory damage; however, they are not a permanent solution for chronic inflammation. One shortcoming of this strategy is that merely stopping continued inflammation is unlikely to reverse lung dysfunction caused by prior inflammatory damage. To investigate the mechanisms of damage associated with chronic TNF-α induced lung inflammation, we utilized a mouse model (SPC-TNF-α) constitutively overexpressing *Tnf*-α in the lungs (Miyazaki et al., 1995). Histological and physiological studies of the SPC-TNF-α lung suggest that it represents the common/converging features of inflammation-induced lung damage in fibrosis and COPD (Fujita et al., 2001; Lundblad et al., 2005; Thomson et al., 2012). COPD is a prevalent multi-systemic disorder with no cure and no efficacious treatment options (Brusselle et al., 2011). We sought to identify reversible physiological processes that are disrupted by the chronic inflammation in COPD and other lung disorders. The SPC-TNF-α model offers unique advantages to do so and may lead to the discovery of novel treatment and prevention alternatives.

Maintaining homeostasis of trace elements (e.g., biometals and minerals) is pivotal for normal physiology; imbalance in any of them may lead to pathological outcomes in humans (Rayman, 2012; Xu et al., 2013). Trace elements play a number of roles which may link their level of activity to the severity of COPD. Copper (Cu), zinc (Zn), and selenium (Se) are all biometals/minerals that regulate redox balance, thereby suppressing oxidative stress. Considering that oxidative stress is involved in the progression of lung inflammation, any disruption of these trace element levels in COPD patients may result in tissue inflammation and damage (Chung and Adcock, 2008; Zuo et al., 2012, 2015). Therefore, monitoring their levels in COPD patients has potential significance when evaluating treatment options. If trace elements are significantly disrupted following chronic inflammation, this pathophysiological process may be important for the therapeutic target of the disease. Currently, trace elements in the clinical setting have failed to establish a consensus regarding the relationship of their serum levels and COPD partly due to the challenges of establishing accurate measurement in the human lungs (Karul et al., 2003; Karadag et al., 2004; Tanrikulu et al., 2011). In order to overcome these barriers, we sought to investigate trace element status in an animal model where we could directly take the measurements from the tissue. These trace elements can then be assessed for their association with the COPD-like lung phenotypes of the SPC-TNF-α model as described previously (Zuo et al., 2014). In this study, we examined the profiles of three key biometals and micronutrients in chronically inflamed tissues. We compared Cu, Zn, and Se contents in the tissues from SPC-TNF-α and wild-type (WT) mice. We reported a marked reduction of Cu levels in the lungs, but not other tissues in our animal model. This coincided with a decreased expression of multiple Cu-responsive genes, including relevant signal factors, Cu-dependent enzymes (proteins requiring Cu as a cofactor), and regulators for Cu homeostasis. These results also revealed a discrepancy between direct Cu quantitation in lung with current clinical approaches that attempt to infer Cu-status from plasma (Kadrabova et al., 1996; Malavolta et al., 2010). Our results suggest that TNF-α-induced chronic lung inflammation results in severe Cu deficiency and such imbalance of Cu homeostasis might contribute to the pathogenesis of chronic inflammatory lung diseases such as COPD.

## Materials and methods

### Animal care and tissue isolation

SPC-TNF-α transgenic mice used in this study demonstrated chronic pulmonary inflammation as well as oxidative stress resulting from the constitutive overexpression of *Tnf*-α in the alveolar epithelial cells (Zuo et al., 2011, 2014). All animal experiments were approved by the Oakland University Institutional Animal Care and Use Committee (IACUC #15065). Aged mice over 16 months old were used in this study. Age-matched transgenic mice (*n* = 7) and WT mice (*n* = 7) were weighed and sacrificed by CO_2_ asphyxiation. Whole blood, heart, lung, liver, spleen, and kidney tissues were collected from each mouse. One half of the tissue was snap frozen by liquid nitrogen for gene expression analysis and the other half was weighed and used for assessment of trace element content.

### Fixation of lungs and quantification of airspace enlargement

The lung was *in situ* fixed based on the method of Braber et al. (2010). In order to prevent the flow of blood into the bases of the lungs, the mice were exsanguinated by an incision to the caudal vena cava (Braber et al., 2010). A cannula was then inserted into the trachea and the left lung was inflated by gentle infusion of cold 4% paraformalin for 5 min. After inflation, the lungs were immersed in fresh fixative for 24 h. The lung lobes were then embedded in paraffin and cut into 5-mm transverse sections. Sections were stained with hematoxylin and eosin (H&E). Enlargement of alveolar spaces was determined by the measurement of mean linear intercept (MLI) using image analysis software (ImageJ) (Luthje et al., 2009).

### Quantification of Cu, Zn, and Se in animal tissues

Three trace elements Zn, Cu, and Se were measured in isolated mouse tissue. Tissues were separated and digested in 100 μL of 70% nitric acid at 80°C until dissolved. The samples were then centrifuged and supernatants were diluted in 1% nitric acid. Trace element content was quantified by an Inductively Coupled Mass Spectrometer (ICP-MS, Perkin-Elmer Nexion 300, Waltham, MA) (Klemm et al., 2011).

### Semi-quantitative RT-PCR in gene expression studies

Lung samples were analyzed by semi-quantitative RT-PCR for expression of genes related to Cu homeostasis and proteins that use Cu as a cofactor. Primer sequences are given in Table 1. Total RNA was isolated from mouse lung tissue using the RNAeasy kit (Qiagen, Hilden, Germany), and an equal amount of RNA was reverse transcribed into cDNA using M-MLV enzyme and random hexamer primers. PCR products were separated by electrophoresis on an agarose gel and quantified using ChemiDoc Touch System (BioRad, Hercules, CA).

**Table 1:** **DNA sequence of primers used to detect gene expression by semi-quantitative RT-PCR**.

	**GENE**	**FORWARD (5′-3′)**	**REVERSE (5′-3′)**
Cu transporter	*Ctr1*	GGGCTTGGTAGAAGTCCGTA	GAAAGTATCCCGTCCCAGCC
	*Ctr2*	CCGCAATCCTAGTCGAGTCC	GTGGTCTGTCCCCTAAAGGC
	*Atp7a*	GTGGGCTGGGAAAGCCG	GTGCTGTGCTCTTCACAAGC
	*Atp7b*	GATGAAAGGACAGACGGCCA	TGCACTGCTCTTCATCCCTG
Cu chaperon	*Ccs*	GAACCATCGACGGCCTAGAG	GCTACAGCACTTATCTGCCCT
	*Atox1*	ACTGCCCGTGTGTGCC	GCCAAGGTAGGAAACAGCCT
	*Cox17*	CAGGGTAGTCGGAGTTTGGG	TCACAAAGTAGGCCACCACG
Cu protein	*Sod1*	AACCATCCACTTCGAGCA	CAATCCCAATCACTCCAC
	*Aoc3*	TGGGTTTTACCCTCACCCCAT	TCCGGTTGCCAAGGTACAAT
	*Lox*	AGGGCGGATGTCAGAGACTA	AATCCCTGTGTGTGTGCAGT
Other genes	*Gapdh*	CCAATGTGTCCGTCGTGGATCT	GTTGAAGTCGCAGGAGACAACC
	*Tnf-α*	TAGCCCACGTCGTAGCA	GGGGTCAGAGTAAAGGGGTC
	*Vegf*	ACTGGACCCTGGCTTTACTG	CTTGCGCTTTCGTTTTTGACC
	*Fak*	AGCTTCAGCCCCAGGAAATC	TGCTGATGAGCTCGCCTAAG

### Statistical analysis

Experimental results are expressed as mean ± SE. Differences between groups were determined by an unpaired two-tailed Student's *t*-test using R software. Statistical significance was achieved when *p* < 0.05.

## Results

### Pathology associated with chronic lung *Tnf*-α overexpression

**Figure 1:**
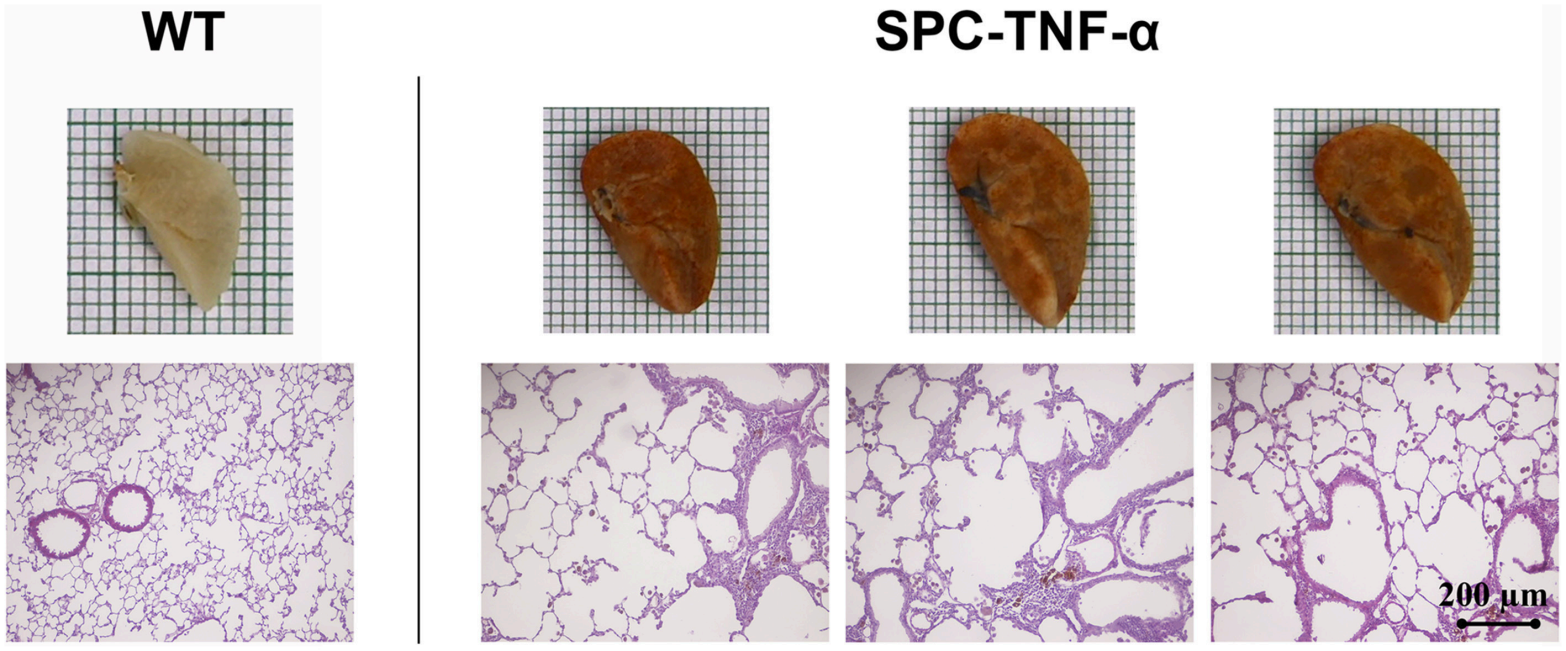
**Representative lung morphology images showing the difference in size and color between H&E-stained lung sections of wild-type and SPC-TNF-α mice**.

**Figure 2:**
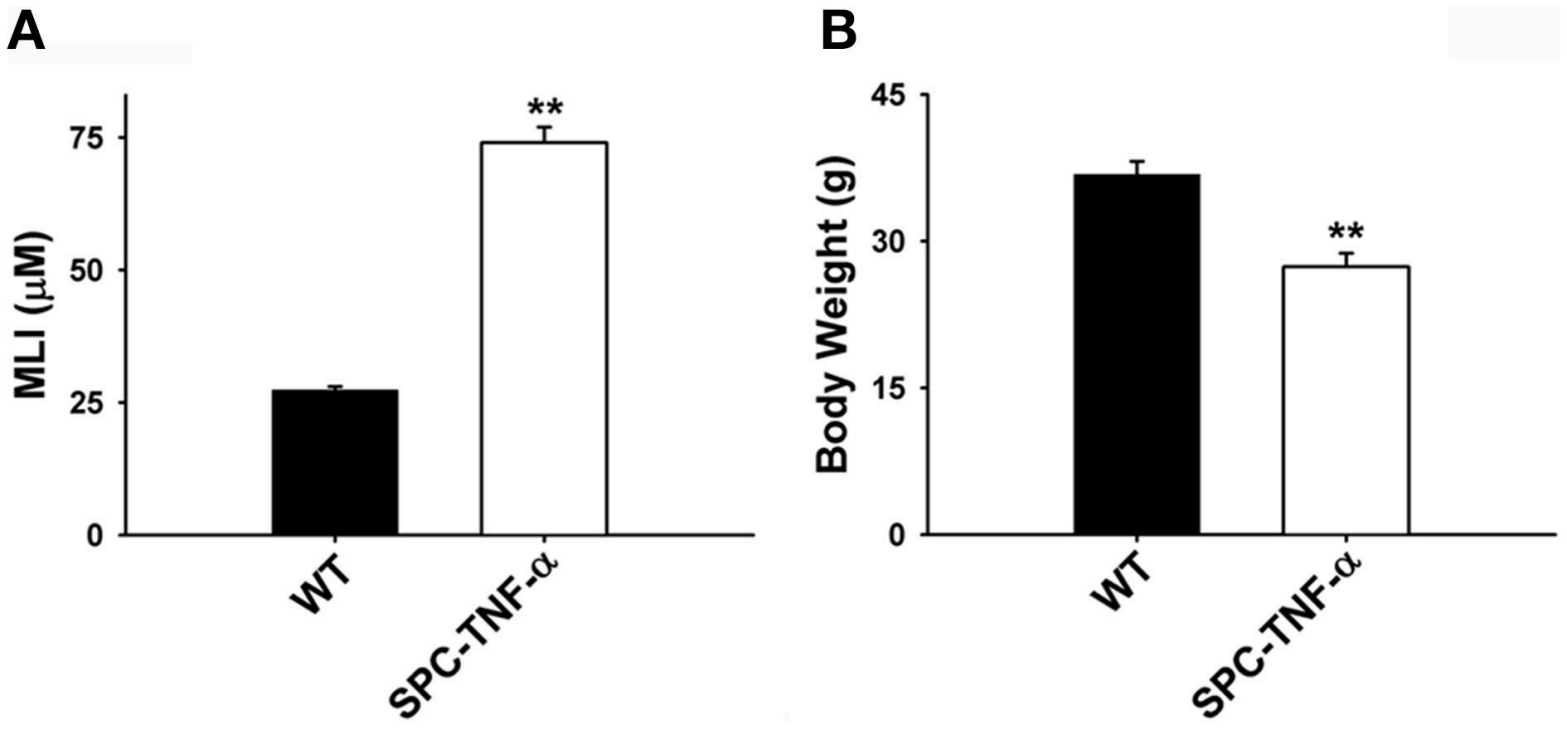
**Mean data comparing the (A) mean linear intercept (MLI) and (B) body weight between wild-type (***n*** = 7) and SPC-TNF-α (***n*** = 7) mice**. Data are expressed as mean ± SE. ^**^Significantly different from control (*p* < 0.01).

Chronic constitutive overexpression of *Tnf*-α in our SPC-TNF-α mouse model led to increased lung volume, as well as color changes (Figure 1). H&E staining of lung tissue sections further confirmed that SPC-TNF-α mice developed signs of COPD and pulmonary emphysema, characterized by abnormal morphology of enlarged air spaces (Figure 1). MLI, a common quantitative measurement of emphysema, was used to quantify the degree of air-space enlargement. MLI increased significantly (27.30 ± 0.69 vs. 73.97 ± 2.94 μm, *p* < 0.01, *n* = 6) while body weight decreased significantly (36.81 ± 1.35 vs. 27.38 ± 1.40 g, *p* < 0.01, *n* = 7) in SPC-TNF-α mice compared to wild type mice (Figures 2A,B). These data demonstrated that mouse lungs with chronic overexpression of *Tnf*-α shares these hallmark features of COPD, consistent with previous reports for mice of various ages (Miyazaki et al., 1995; Fujita et al., 2001; Vuillemenot et al., 2004; Eurlings et al., 2014).

### Homeostasis of Cu is specifically disrupted in the *Tnf*-α transgenic lung

Micronutrients and biometals have been reported to change in response to different types of inflammation. It is known that acutely inflamed tissues can experience decreased Se and Zn, while Cu is often increased in plasma (Prasad, 2009; Hodgkinson and Petris, 2012; Huang et al., 2012). Decreases in levels of biometals and micronutrientss can promote numerous pathologies that may lead to general tissue damage and the progression of COPD (Karadag et al., 2004; Chung and Adcock, 2008). Due to the association of micronutrient/biometal deficiency with processes characteristic of COPD progression, we quantified the levels of Cu, Zn, and Se in the whole blood, heart, lung, liver, spleen, and kidney using ICP-MS (Roman et al., 2014).

**Figure 3:**
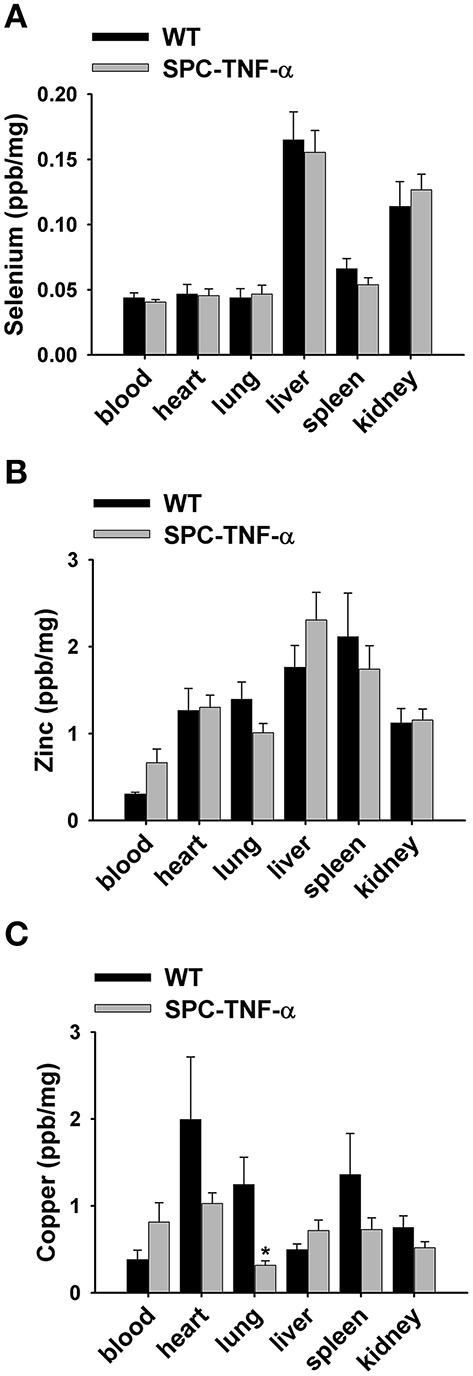
**Mean data showing the concentrations of (A) selenium (***n*** = 7), (B) Zinc (***n*** = 7), and (C) copper (***n*** = 7) in tissues of wild-type and SPC-TNF-α mice**. Mice tissues were isolated and digested, then quantified by ICP-MS. Concentrations were standardized using “wet” weight concentration (ng Se/mg tissue). Data are expressed as mean ± SE. ^*^Significantly different from control (*p* < 0.05).

Among these trace elements, Se concentrations remained approximately constant in all analyzed tissues between SPC-TNF-α and WT mice (Figure 3A). The Zn level in the tissues of SPC-TNF-α and WT mice varied, but the differences were not statistically significant (Figure 3B). Similar to Se and Zn, there were no large differences in Cu concentration in the blood, heart, liver, spleen, and kidney. However, Cu concentration in the lungs significantly decreased (75%, *p* < 0.05) in the SPC-TNF-α mice compared to the WT mice (Figure 3C). Notably, no significant differences in Cu levels were observed in the whole blood of WT and SPC-TNF-α mice.

### Cu-dependent proteins Aoc3 and Lox, Cu transporters *Atp7a* and *Atp7b*, and Cu-responsive signal factors Vegf and Fak are downregulated in the transgenic *Tnf*-α lung

**Figure 4:**
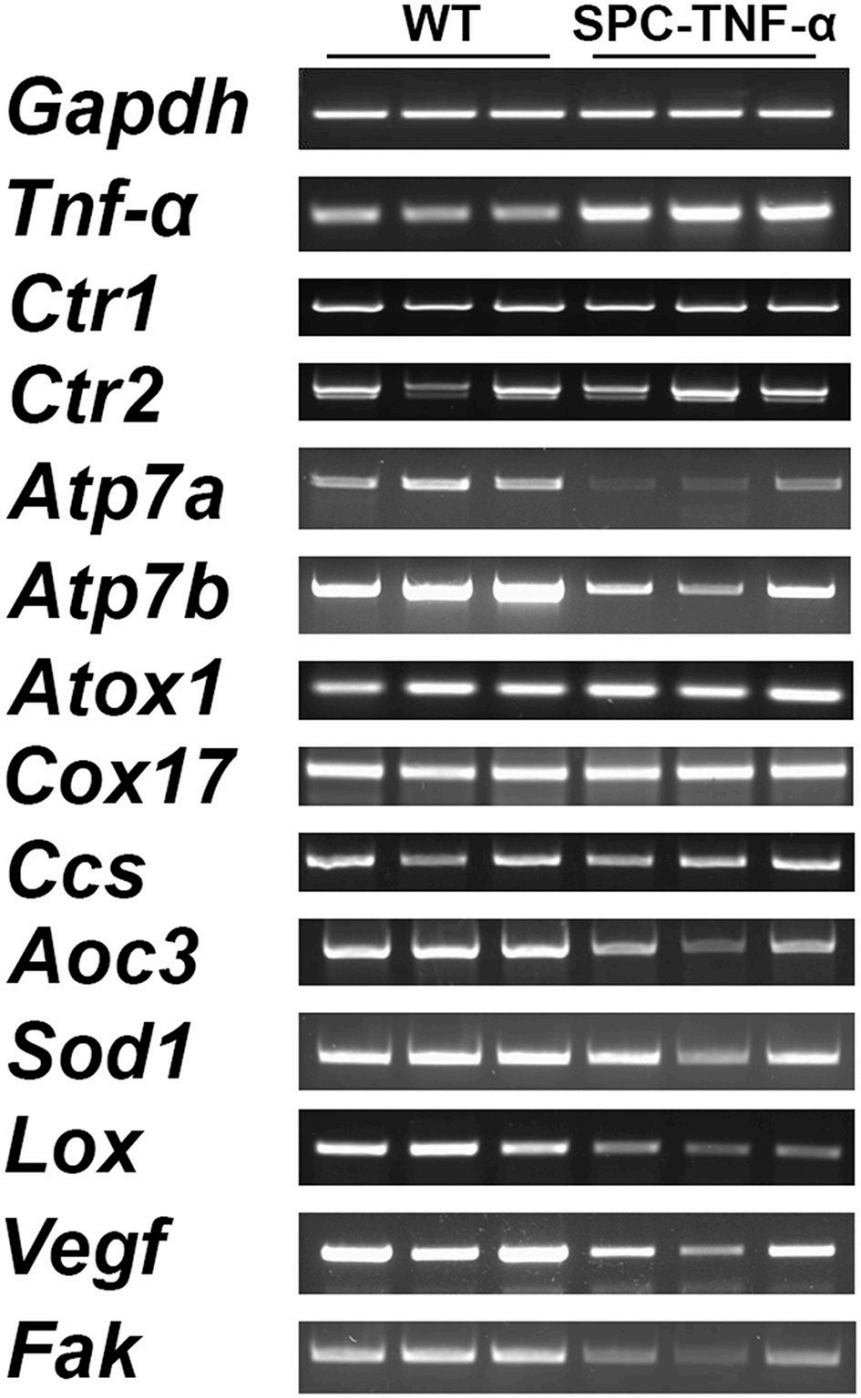
**Representative gel images showing the expression of genes involved in Cu trafficking (transporters and chaperones) and several Cu enzymes critical to immunity and ECM structure analysis in lung tissues from SPC-TNF-α and WT mice**.

**Figure 5:**
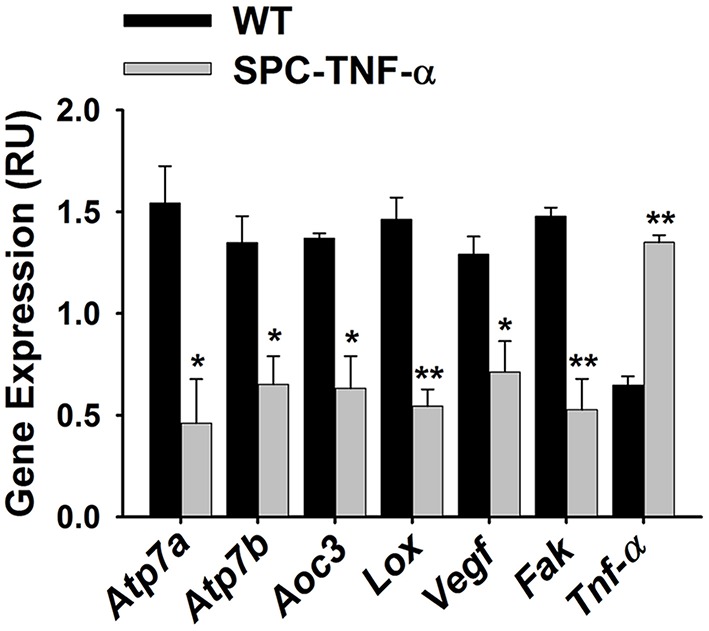
**Bar graph comparing the relative gene expression of WT and SPC-TNF-α lungs for two Cu transporters (***Atp7a*** and ***Atp7b***, ***n*** = 3 each); two Cu enzymes (***Aoc3*** and ***Lox***, ***n*** = 3 each); and two signaling proteins reported to relate to Cu-status (Vegf and Fak, ***n*** = 3 each)**. Expression was quantified by ImageJ based on the gel images. Data are expressed as mean ± SE. ^*^Significantly different from control (*p* < 0.05). ^**^Significantly different from control (*p* < 0.01).

Following our finding that Cu is drastically decreased in the *Tnf*-α transgenic mouse lung, we hypothesized this would result in changes in the expression of Cu associated proteins, such as Cu transporters, Cu chaperones, and Cu enzymes. We examined the gene expression of these enzymes by semi-quantitative RT-PCR (representative gel images are shown in Figure 4). We used the expression level of *Gapdh* as a baseline control and then investigated Cu homeostasis genes including Cu importers, Cu transporter *Ctr1* and *Ctr2* as well as Cu intracellular and efflux transporters ATPases as *Atp7a* and *Atp7b*. Altered expression was only observed with *Atp7a* and *Atp7b*, which were downregulated roughly two-fold (Figure 5). We examined other Cu homeostasis genes, such as antioxidant 1 Cu chaperone (*Atox1*), Cu chaperone for superoxide dismutase (*Ccs*), and cytochrome c oxidase Cu chaperone (*Cox17*), both of which are chaperones involved in intracellular Cu delivery and metabolism. However, the analyzed genes showed no difference in expression between the two groups (data not shown).

Next, we examined changes in the expression of several cuproenzymes: superoxide dismutase 1 (*Sod1*), an antioxidant protein; lysyl oxidase (*Lox*), an extracellular structural protein; and amine oxidase, copper containing 3 (*Aoc3*), a vascular adhesion protein. Among this group, both *Lox* and *Aoc3* gene expressions decreased in the inflamed lungs (Figure 5). Since *Lox* plays an important role in the regulation of the extracellular matrix (ECM) status (Mäki et al., 2005), the decrease of *Lox* in inflamed lungs appears consistent with their observed gross morphological defects (Figure 1).

We also measured the expression of vascular endothelial growth factor (*Vegf*) and focal adhesion kinase (*Fak*). Decreased expression of these two factors is proposed to be involved in lung function changes found in Cu-deficiency-induced emphysema and smoking-induced emphysema (Mizuno et al., 2012; Sakhatskyy et al., 2014). We found that the expression levels of both genes significantly decreased in the inflamed lung (Figure 5).

## Discussion

Lung inflammation becomes more prevalent with increasing age and potentially predisposes elderly to several pulmonary diseases (Canan et al., 2014). A large amount of research has been dedicated to reducing inflammatory cascades; however, little has focused on reducing or reversing the damage caused by chronic inflammation (Chung and Adcock, 2008; Barnes, 2013). The SPC-TNF-α transgenic mouse model mimics the common inflammatory features in human lung pathologies and is a convenient tool for investigating the molecular mechanism of lung inflammation. Our studies evaluated the homeostasis of three important trace elements, Cu, Zn, and Se, in the tissues of transgenic SPC-TNF-α mouse model. We report for the first time that Cu is markedly downregulated by TNF-α-induced chronic inflammation in the lungs. These results show that chronic inflammation-induced Cu-deficiency likely plays a causative role in the progression of lung inflammation.

Proper homeostasis of trace elements such as Cu, Zn, and Se is crucial for normal physiology, as an imbalance of these elements can lead to severe pathologies in mammals (Hodgkinson and Petris, 2012; Rayman, 2012; Xu et al., 2013; Roman et al., 2014; Zheng et al., 2015). Due to the multiple roles of these trace elements, the physiological significance of their deficiencies are numerous and complex. These elements have both direct and indirect roles in inflammation, oxidative stress, and immune responses. Their primary role is as cofactors for metalloproteins, including antioxidants, signal transduction, and transcription proteins (Prasad, 2009; Galli et al., 2012; Roman et al., 2014). The emerging findings of Cu deficiency-induced emphysema have suggested Cu as a paramount contributor in the progression of COPD and other lung disorders (Mizuno et al., 2012). Our results showed that chronic inflammation results in dramatic decreases in lung Cu content (~75%). This suggests Cu deficiency may be a factor in disease progression. Interestingly, Zn and Se, which are often decreased during acute severe inflammation, were unaffected in the lungs or other tissues in our chronic inflammation mouse model. Our analysis also showed that Cu was not significantly altered in other tissues, including the whole blood.

Cu is an essential component of antioxidant enzymes, such as superoxide dismutase (SOD) and catalase (Marklund, 1982). Dietary Cu deficiency affects both innate and acquired immunity (Koller et al., 1987; Percival, 1995; Percival et al., 1995; Munoz et al., 2007). Some studies have shown elevated serum Cu levels in COPD patients (Tanrikulu et al., 2011). Other studies have reported increased Zn and Cu levels in sputum samples from a wide range of lung diseases (e.g., bronchiectasis, cystic fibrosis, asthma, and COPD) (Gray et al., 2010). However, contradictory studies have documented no variation in serum metal concentrations of COPD patients (Karul et al., 2003). These inconsistencies have rendered it difficult to fully understand the status of trace element in COPD lungs as well as other respiratory disorders. Most evaluations rely on analyzing serum/urine/sputum samples, since they are easier to access than the patient tissues. As a result of this limitation, the evaluation and supplementation of minerals and biometals remains unexplored and is not a recommended treatment in COPD (Karul et al., 2003). Therefore, the most ideal alternative is to directly examine the outcomes of chronic inflammation in tissue samples using an animal model. Our SPC-TNF-α mouse model, which displays a clear COPD-like pathological condition, shows a trend of elevation of Cu in the whole blood, yet it is not at significant. In contrast, Cu concentration is markedly reduced in the lungs. This demonstrates that serum samples, often the basis for the clinical diagnosis of Cu deficiency, in this case appears not to accurately reflect tissue Cu status during chronic lung inflammation. Consequently, Cu deficiency may be an undetected occurrence in human lung disorders. This may be a serious concern due to the known general importance of Cu to normal lung function (Mahabir et al., 2007).

**Figure 6:**
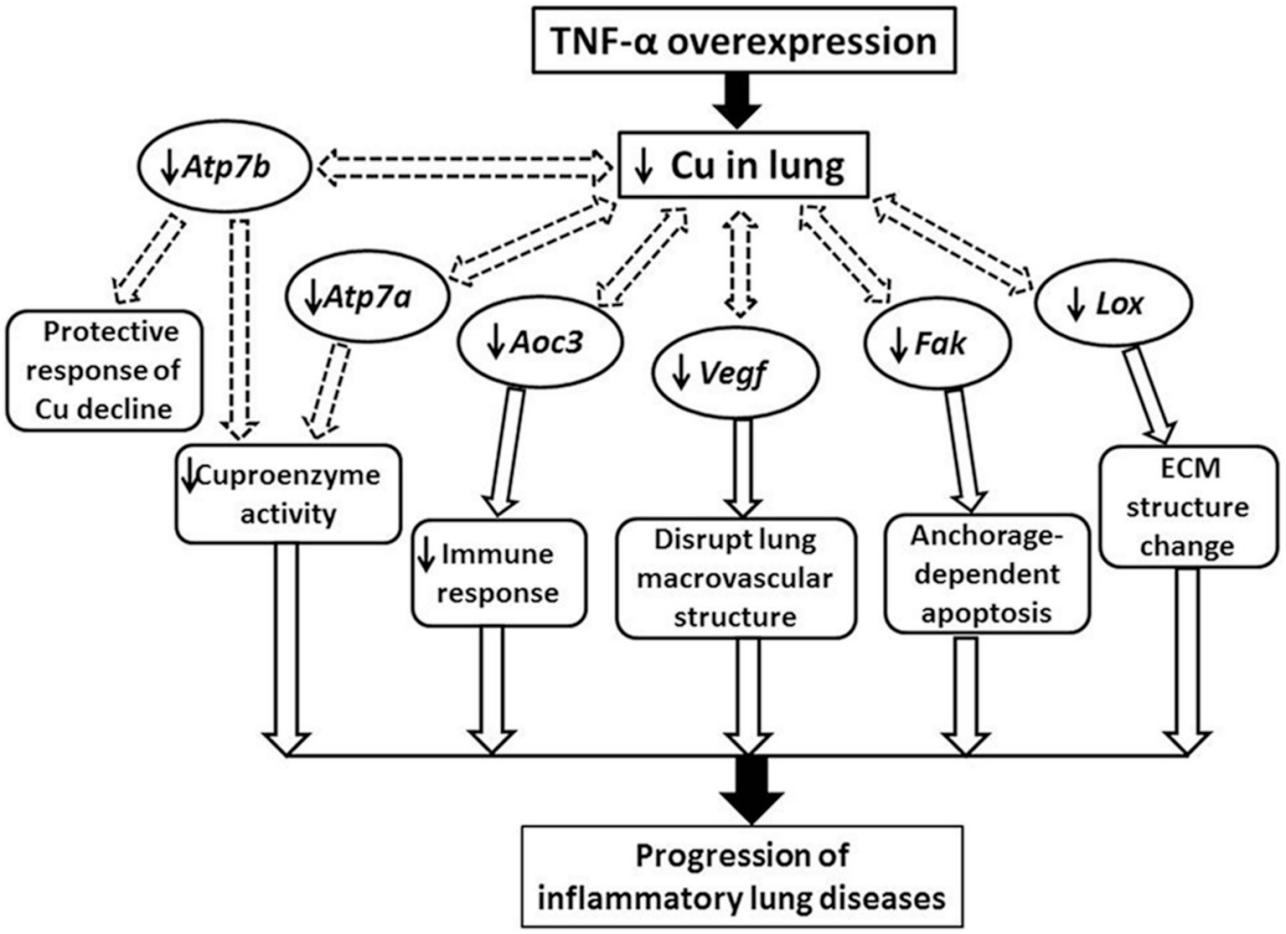
**Schematic illustrating a putative mechanism underlying chronic inflammatory lung diseases involving Cu-deficiency-induced gene expression disruptions in TNF-α lungs**. *Tnf*-α, tumor necrosis factor-α; *Aoc3*, amine oxidase, copper containing-3; *Lox*, lysyl oxidase; *Vegf*, vascular endothelial growth factor; *Fak*, focal adhesion kinase; *Atp7a*, copper-transporting P-type ATPase 7a.

Our results indicated the *Sod1* expression does not differ between lungs with chronic inflammation and WT controls. We also observed that several other Cu proteins are dysregulated due to the lung pathology. Moreover, *Aoc3*, the Cu amine oxidase, is downregulated in the *Tnf*-α overexpressed transgenic lung. *Aoc3* encodes a member of the semicarbazide-sensitive amine oxidase family, which catalyzes the oxidative conversion of amines to aldehydes in the presence of Cu and quinones. As an endothelial adhesion molecule involved in the extravasations of immune cells to sites of inflammation, dysregulation of *Aoc3* is expected to affect immune responses and may be associated with lung pathologies (Figure 6; Dunkel et al., 2014). In addition, another Cu-dependent enzyme gene *Lox* was found to be downregulated in the SPC-TNF-α lung. *Lox* is responsible for the maturation of collagen and elastin (Mäki et al., 2005). *Lox* is crucial for the development of the respiratory system in humans. Transgenic mice with decreased *Lox* expression show pathologies resembling those found in human patients with emphysema and dilated distal airways (Mäki et al., 2005; Kumarasamy et al., 2009). This leads to the possibility that Cu-induced *Lox* deficiency may contribute to the development and progression of emphysema.

Cu homeostasis is associated with cellular copper transporters, including importers and exporters, as well as intracellular endosomal and endoplasmic reticulum transporters. Here we examined the expression of four transporters: *Ctr1* and *Ctr2* (importers); *Atp7a* and *Atp7b* (intracellular transporter and exporter). Surprisingly, Cu importers were not downregulated despite major Cu deficiencies in the lung. Both *Atp7a* and *Atp7b* were downregulated in the SPC-TNF-α lung. Both of these transporters are critical for the provision of Cu to cuproenzymes and the efflux of intracellular Cu (La Fontaine and Mercer, 2007). On the one hand, downregulation of *Atp7a* and *Atp7b* may potentially decrease overall Cu delivery to cuproenzymes and consequently inhibit their activities. On the other hand, downregulation of the transporters *Atp7a* and *Atp7b* is likely a protective response to preserve intracellular Cu during deficiency.

Support for a causative role of decreased Cu in emphysema development has been found in studies of rats where Cu-deficient diets developed emphysema (Mizuno et al., 2012). In those studies, Cu deficiency is reported to result in *Fak* and *Vegf* downregulation. These encoded proteins are critically important for the maintenance of the lung structure. For example, VEGF is involved in maintenance of bronchial and alveolar structures in the lungs (Tang et al., 2004). On the other hand, the loss of FAK function causes anchorage-dependent apoptosis (Mizuno et al., 2012). Our results showed that the expression of *Vegf* and *Fak* is downregulated during Cu deficiency in the constitutive *Tnf*-α overexpressing lungs. This suggests that the Cu deficiency may impair the FAK pathway, leading to cell apoptosis and thus the development of emphysema-like symptoms of SPC-TNF-α lungs (Mizuno et al., 2012). Consequently, we have presented a putative physiological mechanism involving Cu-mediated proteins such as *Atp7a, Aoc3, Vegf, Fak*, and *Lox* in the chronic inflammatory lung diseases (Figure 6). However, not all Cu-related proteins were affected by the severe Cu deficiency in the SPC-TNF-α lung. These included Cu chaperones: *Cox17, Ccs*, and *Atox1*, cuproenzymes: *Sod1* and Cu transporter: *Ctr1 and Ctr2*.

Our SPC-TNF-α mouse model, which displays a clear COPD-like pathological condition, displayed a trend toward an elevation of Cu in whole blood, although this did not reach statistical significance. It is also recommended to supplement individual trace element when needed and avoid the use of parenteral multi-trace element nutrition (Howard et al., 2007; Vanek et al., 2012). Possible mechanisms of Cu supplementation benefit could be the following: (1) restoring critical Cu-dependent enzyme function, and (2) benefitting supraphysiological cupri-enzyme activities, which are observed to be generally beneficial in counteracting various forms of stress and to promote wound healing (Fukai and Ushio-Fukai, 2011; Duncan and White, 2012). To date, Cu supplementation is not a standard treatment for any human lung disorder. However, based on our discussion above, we point to the plausibility of such a regimen. Following our discovery that severe lung pathology is associated with dramatic Cu deficiency, we hypothesize that Cu supplementation can prevent and ameliorate inflammatory damage. Lung-specific Cu supplementation may be an interest of future research.

## Author contributions

Conception and design of research: ZL, LZ. Performed experiments: LL. Assisted with experiments: XG, JM, JS, CC, SX, JK. Analyzed data: LL, LZ, ZL. Interpreted results of experiments: LL, LZ. Contributed reagents/materials/analysis tools: LL, LZ. Prepared figures: LL, LZ. Drafted manuscript: LL, LZ, ZL. Edited and revised manuscript: LZ, ZL. Approved final version of manuscript: LL, XG, JM, JS, CC, SX, JK, LZ, ZL.

## Funding

This work was supported by NIH ES022800 to ZL.

### Conflict of interest statement

The authors declare that the research was conducted in the absence of any commercial or financial relationships that could be construed as a potential conflict of interest.
